# Time to endoscopic vacuum therapy—lessons learned after > 150 robotic-assisted minimally invasive esophagectomies (RAMIE) at a German high-volume center

**DOI:** 10.1007/s00464-022-09754-1

**Published:** 2022-11-07

**Authors:** Seung-Hun Chon, Stefanie Brunner, Dolores T. Müller, Florian Lorenz, Raphael Stier, Lea Streller, Jennifer Eckhoff, Jennifer Straatman, Benjamin Babic, Lars M. Schiffmann, Wolfgang Schröder, Thomas Schmidt, Christiane J. Bruns, Hans F. Fuchs

**Affiliations:** 1 Interdisciplinary Endoscopy Unit, Department of General, Visceral, Cancer, and Transplant Surgery, University Hospital of Cologne, Kerpener Street 62, 50937 Cologne, Germany; 2 Interdisciplinary Endoscopy Unit, Department of Gastroenterology and Hepatology, University Hospital of Cologne, Kerpener Street 62, 50937 Cologne, Germany

**Keywords:** Anastomotic leak, Endoscopic vacuum therapy, Leak management, Upper gastrointestinal surgery, Complication management, Robotic surgery, RAMIE, Esophagectomy

## Abstract

**Objective of the study:**

In esophageal surgery, anastomotic leak (AL) remains one of the most severe and critical adverse events after oncological esophagectomy. Endoscopic vacuum therapy (EVT) can be used to treat AL; however, in the current literature, treatment outcomes and reports on how to use this novel technique are scarce. The aim of this study was to evaluate the outcomes of patients with an AL after IL RAMIE and to determine whether using EVT as an treatment option is safe and feasible.

**Material and methods:**

This study includes all patients who developed an Esophagectomy Complications Consensus Group (ECCG) type II AL after IL RAMIE at our center between April 2017 and December 2021. The analysis focuses on time to EVT, duration of EVT, and follow up treatments for these patients.

**Results:**

A total of 157 patients underwent an IL RAMIE at our hospital. 21 patients of these (13.4%) developed an ECCG type II AL. One patient died of unrelated Covid-19 pneumonia and was excluded from the study cohort. The mean duration of EVT was 12 days (range 4–28 days), with a mean of two sponge changes (range 0–5 changes). AL was diagnosed at a mean of 8 days post-surgery (range 2–16 days). Closure of the AL with EVT was successful in 15 out of 20 patients (75%). Placement of a SEMS (Self-expandlable metallic stent) after EVT was performed in four patients due to persisting AL. Overall success rate of anastomotic sealing independently of the treatment modality was achieved in 19 out of 20 Patients (95%). No severe EVT-related adverse events occurred.

**Conclusion:**

This study shows that EVT can be a safe and effective endoscopic treatment option for ECCG type II AL.

Esophageal malignancy is the sixth most common cause of cancer-associated deaths worldwide [1]. The current curative standard treatment is multimodality therapy combined with transthoracic esophagectomy and 2-field lymph node dissection. However, esophagectomy is a complex surgical procedure associated with substantial morbidity and mortality [2–4]. The evolution towards minimally invasive techniques has led to reduced cardiopulmonary complication rates and reduced pain after esophagectomy.

The acceptance of robot-assisted minimally invasive esophagectomy (RAMIE) is mostly attributed to improved clinical outcomes through better vision and dissection capabilities [5]. Although RAMIE has shown to further reduce anastomotic leak rates compared to the hybrid minimal invasive approach, anastomotic leaks (AL) are still among the most critical adverse events [6, 7]. The impact of AL is severe and can increase hospital stay, morbidity, and have a negative impact on the long-term outcome [8, 9].

Over the last few decades, the management of AL has shifted from surgery to a variety of endoscopic interventional techniques [10–15]. Using stents is an established therapeutic option, and endoscopic vacuum therapy (EVT) has become a promising alternative [16, 17]. Several studies have demonstrated high closure rates of approximately 90% [18]. EVT can be used in two different ways: intraluminal positioning of the sponge in the esophageal lumen and intracavitary placement to treat a para-esophageal wound cavity [19]. Due to the lack of comparative studies, no firm conclusions concerning the superiority of one treatment method can be drawn at this point. Overall treatment time can be short, but the sponge-system needs to be changed frequently, so repeated endoscopies are necessary.

However, reports of the use of EVT for AL especially in large cohorts and after a minimally invasive i.e., robotic techniques do not exist. Therefore, our goal was to evaluate the safety and feasibility of EVT in patients with an Esophagectomy Complications Consensus Group (ECCG) type II AL after an Ivory-Lewis (IL) RAMIE procedure at a European High-Volume Center.

## Materials and methods

### Patient cohort

A retrospective chart review was performed at the Department of General, Visceral, Cancer, and Transplant Surgery in cooperation with the Interdisciplinary Endoscopy Unit at the University Hospital Cologne. Patients that underwent an oncological IL RAMIE with transthoracic gastric pull-up reconstruction and presented with an ECCG AL type II were included in the analysis.

Esophageal AL is defined according to the ECCG classification as a full thickness GI defect involving esophagus, anastomosis or staple line irrespective of presentation or method of identification. The leak were further classified as, Type I: local defect requiring no change in therapy or treated medically or with dietary modification; Type II: localized defect requiring interventional but not surgical therapy; Type III: localized defect requiring surgical therapy [20].

Data were retrieved from our prospectively maintained hospital database “Orbis” (version 08,043,703; Agfa HealthCare N.V., Belgium) and from our prospectively maintained endoscopic database “Clinic WinData” (version 8.06; E&L medical system GmbH, Erlangen, Germany). The following information was collected: demographic and clinical patient characteristics, details of the disease, leak characteristics, time to EVT, duration of EVT, and follow-up treatments.

### Statement of ethics

The manuscript was submitted to the local ethics committee, which stated that we are exempt from applying for ethical approval—under German law, no separate ethics application and statement of ethical approval by the local ethics committee is required for performing purely retrospective clinical studies.

### Surgical approach

Ivor-Lewis robotic-assisted minimally invasive transthoracic esophagectomy (IL RAMIE) using a gastric conduit for reconstruction of the gastrointestinal passage was performed either completely robotic (robotic gastrolysis, robotic transthoracic esophagectomy) or in hybrid from, totally minimally invasive (laparoscopic gastrolysis, robotic-assisted transthoracic esophagectomy) using the Da Vinci X or the Da Vinci Xi System (Da Vinci X/Xi system, Intuitive Surgical Inc. Sunnyvale, CA, USA). An updated robotic technique using a 28 mm circular stapler (Medtronic, Covidien, EEA 28 mm DST Circular Stapler, Medtronic GmbH Meerbusch/Germany) and indocyanine green for angiography of the gastric conduit and anastomotic region was implemented in 2019. In cases with a narrow esophagus, a 25 mm circular stapling device was used. We have published our technique previously [21].

### Leak detection and treatment pathway

Several diagnostic criteria are available for AL detection. First of all, clinical signs were evaluated. The symptoms for AL range from no signs to fulminant sepsis, however, patients often present with arterial fibrillation, fever, chest pain or dyspnea. Additional blood tests were performed daily. A high level of blood inflammatory biomarkers (C-reactive protein (CRP), procalcitonin, and white blood cell counts) are good indicators for an AL. Based on clinical symptoms, overall state of the patient and the biochemical analysis the choice for additional diagnostics was made. In line with the current literature, a CT scan and EGD are the standard in our clinic [22]. Patients with a suspected AL received a flexible video esophagogastroduodenoscopy (EGD) (e.g., GIF-H190; GIF-XP180N; Olympus Medical Systems, Tokyo, Japan) with photo and video documentation. All procedures were performed under sedation with propofol (e.g., Fresenius Kabi Germany GmbH) or using general anaesthesia, if performed at the intensive care unit. EGD was performed by a board certified surgical attending, with expertise in surgical endoscopy at our interdisciplinary surgical endoscopy unit, which is a certified national reference center for upper gastrointestinal cancers. The choice between EVT and surgical revision as treatment options was based on an evaluation of defect size, perfusion of the conduit, postoperative day and clinical state of the patient. Cases of early AL combined with conduit necrosis usually underwent surgical revision, while cases of late AL were treated endoscopically. However, the postoperative day was not used as a strict cut off criteria. Previous studies have shown that patients with an early anastomotic leak benefit from surgical revision [23].The decision whether the AL is managed surgically or endoscopically is made by the operating surgeon in consensus with the endoscopist who has completed surgical training and is familiar with esophageal cancer surgery. The EGD is either directly shown to the respected surgeon or video recorded. Revisional cases are further discussed in an interdisciplinary clinical conference of the surgical department, where the EGD and CT scans are shown, and the clinical state of the patient is presented.

The CT scan was performed to detect mediastinal fluid collections and an additional drain was placed if needed. An empiric antibiotic and antifungal therapy was administered immediately. After successful EVT, defined as complete closure of the defect detected by EGD, sufficient keeping of air and fluids of the gastric conduit, the EVT was removed. Additionally, a swallowing study was performed to confirm the successful treatment. The feeding was slowly started after completion with liquids and gradually increased to a normal diet within a few days.

### Endoscopic vacuum therapy

**Fig. 1:**
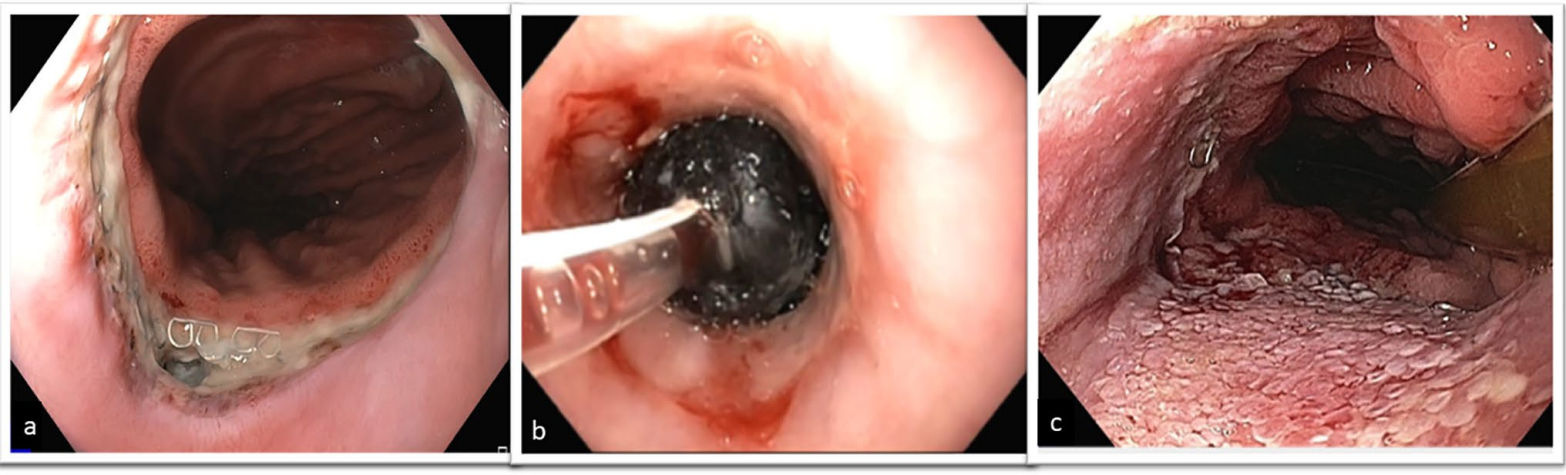
**a** Endoscopy showing a leak of the esophagogastrostomy before EVT treatment. **b** Endoluminal placement of EVT. **c** Sealed anastomotic leak with vital granulation tissue

An open-pore polyurethane sponge (EsoSponge®; Braun Medical, Melsungen, Germany) was adapted in size to correspond to the extent of the leakage. The sponge was then inserted into the para-esophageal wound cavity (intracavitary) or into the esophageal lumen (intraluminal) using an overtube (Fig. 1). The placement of the sponge was performed under continuous endoscopic vision, and the area of the AL was completely covered by the Sponge. The connected tube was then repositioned to the nasal cavity. Afterwards, a defined negative pressure of 125 mm of mercury (mmHg) was applied using an electronic vacuum pump (e.g., VivanoTec®, Hartmann AG, Germany). The sponge was removed endoscopically every 3–5 days and replaced if needed. However, if the patients’ clinical status or the blood inflammatory biomarkers worsened the endoscopy and EVT change was performed immediately. Successful completion of EVT was defined as complete closure of the defect detected by EGD and sufficient keeping of air and fluids of the gastric interpona. Hence the treatment was stopped. In our manuscript we described four patients with a persisting AL. Due to a persisting leakage and insufficient closure of the anastomosis, the placement of a SEMS was indicated. SEMS placement was performed if no healing progress was seen over a course of three EVT changes.

### Additional treatment

A triple-lumen diverted nasogastric feeding tube (Freka® Trelumina, Fresenius Kabi Germany GmbH) was endoscopically placed additionally right before the insertion of the EVT-device (EsoSponge®). The endoscopist made sure that the triple-lumen nasogastric feeding tube was positioned on the opposite side of the anastomotic leak so that the parallel introduced EVT had full contact to the area. The gastric tube of the triple-lumen nasogastric feeding tube was used to decompress the gastric conduit and to evacuate gastric and duodenal reflux. The jejunal tube of the triple-lumen nasogastric feeding tube secured the enteral nutrition of the patient and remained at least until the leak was fully resolved or longer if needed. We routinely place a triple-lumen nasogastric feeding tubes in addition in every patient receiving an EVT for securing an enteral feeding. We started feeding patients through the tripple-lumen diverted nasogastric feeding tube immediately after the endoscopy and started with 20 ml/h of Fresubin.

### Statistics

Statistical comparison and analysis were performed using Fisher’s exact test for nominal data, and student’s *t*-test for continuous data. A *p*-value of less than 0.05 was considered statistically significant. Continuous variables are presented as means and range. Categorical data are presented as numbers and percentages. Data were analyzed by GraphPad Software (San Diego, California, USA) and Microsoft Excel Version 2013 for Windows (Microsoft Corp, Redmond, WA).

## Results

From April 2017 until December 2021 a total of 157 patients underwent an IL RAMIE for cancer at our clinic. No patients showed a type I leak, 21 patients developed an ECCG type II AL (13.4%) and 8 patients developed an ECCG type III AL (5.1%). Oncological and demographic data of patients with and without an AL are depicted in Table 1. The mean BMI was 25.35 kg/m^2^ (range 18.52–33.90 kg/m^2^) in the group of patients with type II compared to a mean BMI of 25.57 kg/m^2^ (range 15.62–35.35 kg/m^2^) in the cohort without any leak, which constitutes no statistically significant difference.

**Table 1:** Patients’ characteristics, statistical significance set at *p* ≤ 0.05

	Overall cohort without leak		ECCG type II anastomotic leak		ECCG type III anastomotic leak		*P* value
	Total /mean	(%/range)	Total /mean	(%/range)	Total /mean	(%/range)	
Patients	128	100	20	100	8	100	–
Age (years)	62.3	41–80	63.7	54–72	64.4	46–80	0.8060
Male/Female	101/ 27	78.9 / 21.1	19 / 1	95.0 /5	7 / 1	87.5 / 12.5	0.4828
ASA	1,9	I-III	2	I-III	2	I-III	
ECOG	0.28	0–1	0,47	0–2	0,62	0–2	
Pathology							
Adenocarcinoma	103	80.5	17	85	7	87.5	1
Squamous cell carcinoma	23	18	3	15	1	12.5	1
Other	2	1.5	0	0	0	0	–
Neoadjuvant Chemotherapy							
None	17	13.3	3	15.0	3	37.5	0.3045
CROSS	67	52.3	8	40.0	4	50	1
FLOT	42	32,8	9	45.0	1	12.5	0.2008
Other	2	1,6	–	0	0	0	–

### Endoscopic vacuum therapy and postoperative course

All cases (21 patients) with an ECCG type II AL received EVT as a first line therapy. Two patients died prior to the completion of endoscopic therapy: one of sepsis with an unknown focus. before EVT therapy could be successfully terminated. One patient was infected with COVID 19 on POD 3 and transferred to ICU for better surveillance. During the clinical course, the patient developed an anastomotic leak which was treated with EVT. A sepsis related to the AL was not found. Additionally, as the COVID 19 pneumonia progressed multi organ failure and especially a distinctive respiratory insufficiency was seen. The patient died on POD 26 respiratory insufficiency due to the COVID 19 pneumonia. Therefore, the latter patient was excluded from the study cohort. Two patients in our cohort received a combined therapy with a hybrid stent, which combines endoluminal endoscopic vacuum therapy with SEMS treatment (VACStent®).

With EVT as mono-therapy, a closure rate was achieved in 15 out of 20 patients (75%). Placement of a SEMS after EVT was performed in 4 patients due to persisting AL. Overall success of anastomotic sealing independently of the treatment modality was achieved in 19 out of 20 Patients (95%).

ECCG Type II AL was diagnosed at a median of 8 days post-surgery (range 2–16 days). The mean duration of EVT was 12 days (range 4–28 days) with a mean of two endoscopic sponge changes (range 0–5 changes). No patient with an initial ECCG type II needed additional surgical therapy. The median postoperative length of stay was 24 days (range 16–59 days) for patients with a type II leak compared to a median of 15 days (range 10–123 days) for patients with no AL. More details about the postoperative course are depicted in Table 2.

**Table 2:** Postoperative course of the study cohort

	Overall cohort without leak		ECCG type II anastomotic leak		ECCG type III anastomotic leak		*P* value
	Total/median	(%)/range	Total/median	(%)/range	Total/median	(%)/range	
Patients	128	100	20	100	8	100	–
Intensive care unit							
Readmission	12	9.4	6	30.0	6	75	0.0923
Reintubation	4	3.1	4	20	4	50	0.2089
Duration of stay	2	1–65	10.05	2–44	8.5	5–112	0.1887

	Overall cohort		ECCG type II leak		ECCG Type III leak		
Clavien Dindo Classification							
IIIa	35	27.3	14	66.6	0	0	0.0022
IIIb	7	5.5	0	0	5	62.5	0.0005
IVa	6	4,7	4	19	2	25	1
IVb	0	0	1	4.8	1	12.5	0.4828
V	1	0.8	1	5	0	0	1

## Discussion

In this study, we present the clinical outcomes of EVT in the management of ECCG type II AL after IL RAMIE at our institution between 2017 and 2021. Our study aimed to report our method of application of EVT for anastomotic leak in patients that underwent a minimally invasive esophagectomy. The analysis showed that EVT is both safe and technically feasible. Additionally, we demonstrated excellent clinical outcomes and low morbidity of this endoscopic treatment modality for this patient cohort that includes all patients who underwent IL RAMIE at our institution.

While an open or hybrid minimally invasive transthoracic esophagectomy is still considered the gold standard for the treatment of esophageal cancer in many centers [24], minimally invasive robotic operating techniques are becoming more popular and depict the standard of care at our high-volume center. While an open technique may be associated with higher rates of postoperative complications according to recent literature, the use of RAMIE may reduce the negative impact without compromising oncological outcomes [7, 25]. Despite the lower morbidity rate of RAMIE, the occurrence of an AL is still one of the most feared life-threating complications. Timely and appropriate treatment is crucial in the management of esophageal AL [8, 9].

Our study cohort showed an ECCG AL type II in 21 of 157 patients (13.4%) after IL RAMIE. This corresponds to the data by van der Sluis et al., Pointer et al., and Egberts et al., who report a leak rate of 11, 13.5, and 13.2%, respectively [26–28]. Compared to the studies of the DCDP Database and the ESO Benchmark database which report an AL rate of 15 and 15.9%, IL RAMIE showed a lower AL rate. This suggests that IL RAMIE may reduce the surgical trauma and can lead to better postoperative outcomes. A large nationwide data analysis supports these findings [29].

The management of AL represents a clinical challenge with an associated risk of mortality and morbidity [30]. One minimally invasive endoscopic treatment approach for this diagnosis is the use of EVT [14]. Several studies have demonstrated high closure rates of approximately 90% with a mortality rate of 10% [18]. In our cohort, all 21 patients with an ECCG type II leak received EVT. One patient died of unrelated Covid-19 pneumonia and was excluded from the study cohort. Successful treatment was observed in 15 of 20 patients (75%) which is in line with our earlier study in which we reported a rate of 71.5% [14, 15]. Moreover, our data showed a similar mean treatment duration of 12 days (4–28 days) as our earlier study and, compared to a recently published register study by Richter et al., a shorter treatment time [15, 31]. The rate of clinical success with this short treatment duration suggests promising results compared to previous studies that focused on alternative endoscopic treatment options for AL such as SEMS treatment [13, 16]. However, the results of the ongoing phase 2 randomized trial (ESOLEAK) that compares EVT with SEMS for the treatment of AL after IL esophagectomy is expected to give us an indication of whether this trend is significant [32].

EVT can be applied in two different ways—intraluminal positioning of the sponge or intracavitary placement to treat a para-intestinal wound cavity [19]. In our series, all patients (*n* = 20) received an intraluminal EVT as primary treatment. In 6 out of 20 patients, the treatment method was switched from intraluminal to intracavitary because a large wound cavity developed during EVT treatment. Three of these received a SEMS as a final treatment due to the unsuccessful closure of intracavitary EVT treatment. An explanation for this may a need for additional treatment options; EVT alone may not be sufficient to treat complex and large wound cavities. Surgical or radiological drainage of any deep thoracic collections, antibiotics, and antifungal therapy should be introduced additionally in the management of these patients. So far we cannot formulate a recommendation for the ideal position of the sponge, and more studies are required to clarify the influence of the sponge position on the healing process.

Preclinical basic research focusing on the underlying mechanisms of EVT is still very limited. Morykwas et al. showed in a porcine model that an effective increase of wound blood flow was reached at a negative pressure of − 125 mmHg, which lead to optimal oxygenation, neoangiogenisis and elaboration of growth factors and consequently to a faster tissue granulation [33]. Contrary to this, Jung et al. presented a clinical trial in which they achieved a sealing rate of 78.3% in an AL subgroup analysis for the mono-therapy of EVT with a pressure between − 20 and − 50 mmHg [34]. We have used − 125 mmHg since we introduced this treatment option in our clinic [14]. Since then, we have observed that the treatment with − 125 mmHg is sufficient for sealing the leak and achieved sufficient granulation of the wound bed. Using this negative pressure, we had less technical complication in terms of dislocation of the sponge or a blocked tube. While these are promising results, a larger patient cohort is needed to verify our results concerning the ideal negative pressure.

Another factor for which we still lack enough evidence to draw firm conclusions is the question of when to exchange the sponge-system. The accepted time frame varies between 2 and 3 days. At our institution, sponge-systems were renewed every 3 to 4 days. This interval was chosen based on the availability of the endoscopy service for routine procedures on weekends. Consequently, in our study the sponge-system may have been exchanged too late. On the other hand, our clinical results confirm our treatment pathway. Overall, the efficacy of leak closure might have been even higher if we had exchanged it more often, and the optimal exchange interval has yet to be determined.

Laukoetter et al. reported that EVT-associated complications occurred in 4.1% of all interventions and with minor bleeding in 1.3% of the patients [35]. They reported two cases of fatal bleeding related to the rupture of adjacent cardiovascular structures. Minor bleeding can often be observed when the sponge is removed. To reduce the risk of this complication, we switched off the vacuum pump 2 hours before the extraction and moistened the sponge. These precautions may have contributed to the fact that no serious adverse events occurred during the EVT treatment. Additionally, the mortality rate of our cohort of patients with an AL was lower than in previously published studies (9.5% versus 26%) [36]. The reason for these positive effects could be the minimally invasive robotic surgical approach combined with a standardized minimally invasive endoscopic treatment with EVT. While these are promising results, a larger patient cohort is needed to verify our results.

Based on the results of our study, EVT seems to be a safe and feasible procedure for treating AL as there were no serious adverse events associated with the application of the device itself in our cohort.

Regarding the retrospective design, our study has certain limitations, including a lack of a comparable cohort with alternative endoscopic treatment options and a limited number of included patients. We included all patients from the very first IL RAMIE case at our institution, a fact that constitutes the inclusion of a learning curve. According to published learning curves of RAMIE and our own published data, this affects at least the first 20 patients in this collective [21, 37]. Some may see this fact as a limitation, whereas we believe this is a strength of our study. Reporting the results of an entire, standardized collective from a single high-volume center is important and insightful. Overall success rate of anastomotic sealing with purely endoscopic technology in ECCG type II AL independently of the treatment modality was achieved in 95%, a fact that underlines the effectivity of our treatment pathway.

In conclusion, our study demonstrates that using EVT for ECCG type II AL is safe und feasible. No patient required surgical reoperation and no patient died due to EVT-related complications.
